# The Heart–Brain Axis: Atrial Cardiomyopathy and Brain Health

**DOI:** 10.1016/j.jacbts.2026.101628

**Published:** 2026-07-08

**Authors:** Maria Lembo

**Affiliations:** Department of Advanced Biomedical Sciences, Federico II University of Naples, Naples, Italy

**Keywords:** cerebral microinfarcts, cerebral small vessel disease, left atrial strain, proteomics, white matter hyperintensity volume

**Figure:**
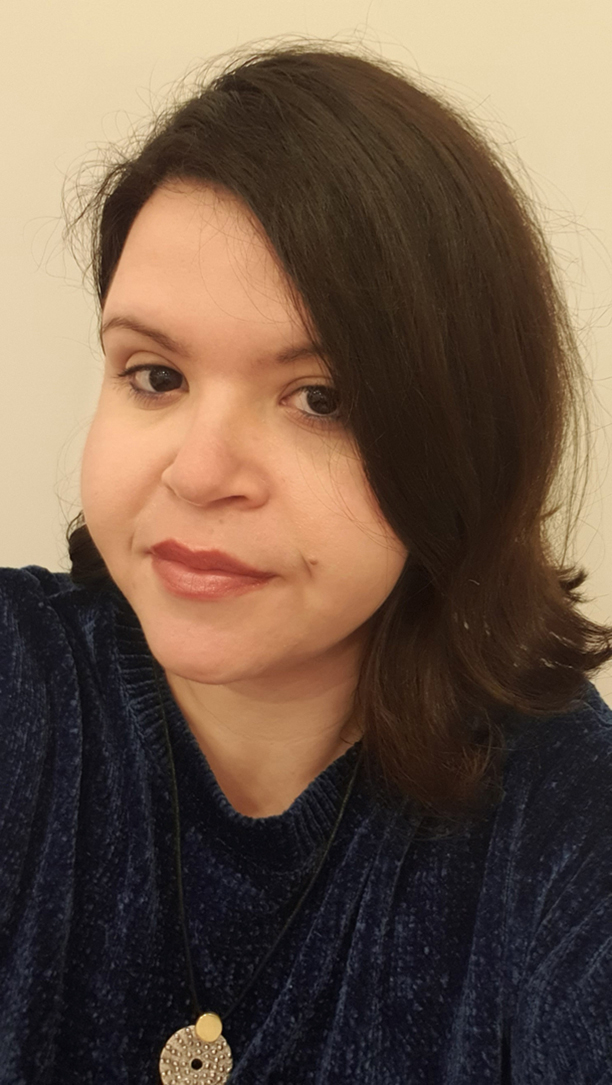

For decades, cardiovascular and neurological diseases have been investigated as largely independent conditions. However, growing evidence suggests that this traditional separation may be overly reductionist. Cardiovascular dysfunction appears capable of profoundly influencing cerebral health, and subtle pathological alterations within the heart may contribute to progressive neurological injury long before overt clinical manifestations emerge.^1^^,^^2^

The acknowledgement of the “heart–brain axis” is reshaping contemporary understanding of both cardiovascular and neurodegenerative disease. It represents a complex biological relationship involving vascular regulation, inflammatory signaling, endothelial integrity, and systemic metabolic processes. Indeed, diseases traditionally considered of cardiovascular relevance may have important neurological consequences, particularly in aging populations.^1^, ^2^, ^3^

The study by Tan et al^4^ investigating the relationship between left atrial dysfunction and cerebral small vessel disease provides important insights into this emerging paradigm. The study examined patients without atrial fibrillation and analyzed the association between left atrial mechanical function and markers of cerebral small vessel disease using advanced imaging and plasma proteomic profiling. Specifically, the researchers evaluated left atrial strain parameters, including left atrial reservoir strain and left atrial conduit strain (LAScd), alongside neuroimaging findings such as cerebral microinfarcts and white matter hyperintensities by magnetic resonance imaging (MRI). These brain abnormalities are widely recognized as major contributors to vascular cognitive impairment, dementia, and age-related cognitive decline.^5^^,^^6^

According to these findings, impaired left atrial function was significantly associated with structural cerebral injury even in the absence of overt atrial fibrillation. Reduced left atrial reservoir strain and LAScd correlated with cerebral microinfarcts, while LAScd was additionally associated with increased white matter hyperintensity volume identified by MRI.^4^

These observations suggest that atrial cardiomyopathy itself may represent an independent pathological entity capable of influencing cerebral integrity, even in the absence of atrial fibrillation, thus implying the possibility to develop cardiac-induced neurological damage, which seems to be independent of traditional thromboembolic stroke pathways known to be linked to atrial fibrillation.^7^

This challenges traditional assumptions and expands the understanding of cardiovascular contributions to neurodegenerative disease.

The importance of these findings lies not only in the demonstrated association but also in the proposed biological mechanisms underlying this heart–brain interaction. Through plasma proteomic profiling of more than 1,400 circulating proteins, researchers identified inflammatory and vascular pathways potentially mediating the relationship between atrial dysfunction and cerebral injury. Among the proteins identified, TNFRSF11A and IGFBP-4 emerged as particularly relevant because of their strong mediation effects and high degree of connectivity within protein interaction networks. These biomarkers are involved in inflammatory regulation, extracellular matrix remodeling, vascular integrity, immune signaling, and cellular proliferation. Their identification reinforces the hypothesis that chronic systemic inflammation and vascular dysfunction may constitute shared pathological mechanisms linking cardiovascular disease and cerebral degeneration.^8^

Indeed, inflammation has long been recognized as a major driver of cardiovascular remodeling. In atrial cardiomyopathy, inflammatory pathways promote fibrosis, endothelial dysfunction, and structural remodeling of cardiac tissue. Similarly, chronic neuroinflammation is increasingly considered a key factor in the progression of cerebral small vessel disease and dementia.^8^^,^^9^ The overlap between these inflammatory pathways suggests that cardiovascular and neurological disorders may arise from interconnected systemic processes rather than isolated organ-specific abnormalities.

The translational implications of these findings are substantial. Dementia currently represents one of the greatest global public health challenges, with rapidly aging populations placing increasing pressure on healthcare systems worldwide. Despite advances in neuroscience, effective treatments for neurodegenerative diseases remain limited, and prevention has become a central strategy in reducing the future burden of cognitive impairment.^10^

The recognition that cardiovascular dysfunction may contribute directly to cerebral degeneration suggests that cardiovascular prevention, controlling risk factors and promoting lifestyle interventions, might also serve as neurological prevention.^10^ Cardiovascular risk factors, particularly arterial hypertension, induce profound structural and functional alterations of the cardiovascular system, including left atrial remodeling, while simultaneously contributing to cerebral injury. Advanced neuroimaging studies using diffusion tensor imaging and MRI fiber-tracking have demonstrated white matter microstructural alterations in hypertensive patients even in the absence of abnormalities detectable by conventional neuroimaging, further supporting the concept of subclinical cardiovascular-mediated brain injury and emphasizing the importance of early prevention.^6^

In addition, another important aspect is related to the growing need for interdisciplinary collaboration between cardiology and neurology. As understanding of the heart–brain axis evolves, neurological assessment may become increasingly relevant in cardiovascular practice, while cardiovascular evaluation may acquire greater importance in patients with cognitive impairment or early neurodegenerative disease. Future clinical models will likely require more integrated approaches capable of simultaneously addressing cardiovascular and neurological risk.

Equally important is the study's methodological framework, reflecting the growing transition toward precision medicine. The integration of advanced cardiac imaging, neuroimaging, and large-scale proteomic profiling represents a powerful multidimensional approach capable of identifying mechanistic pathways linking organ systems previously investigated in isolation. Such strategies may facilitate earlier risk stratification, biomarker discovery, and development of targeted therapeutic interventions.

Several limitations deserve consideration. The cross-sectional design does not allow definitive causality, and longitudinal studies will be necessary to determine whether atrial dysfunction directly drives cerebral injury progression. Furthermore, whether specific cardiac phenotypes, having as a common denominator atrial dysfunction, may be more susceptible to cerebral injury remains to be established.

Nonetheless, the evidence presented by this study strengthens the concept of a biological interplay in the heart–brain axis. Understanding this relationship may profoundly influence future diagnostic models, therapeutic targets, and preventive medicine. Ultimately, the growing convergence between cardiology and neurology suggests that preserving cardiovascular health may also be essential for preserving cognitive function and healthy brain aging.

## Funding Support and Author Disclosures

The author has reported that she has no relationships relevant to the contents of this paper to disclose.
